# Crystal structure of bis­[*S*-hexyl 3-(4-methyl­benzyl­idene)di­thio­carbazato-κ^2^ *N* ^3^,*S*]nickel(II)

**DOI:** 10.1107/S2056989015000328

**Published:** 2015-01-14

**Authors:** M. B. H. Howlader, M. S. Begum, M. C. Sheikh, R. Miyatake, E. Zangrando

**Affiliations:** a Department of Chemistry, Rajshahi University, Rajshahi-6205, Bangladesh; b Department of Applied Chemistry, Faculty of Engineering, University of Toyama, 3190 Gofuku, Toyama 930-8555, Japan; c Center for Environmental Conservation and Research Safety, University of Toyama, 3190 Gofuku, Toyama 930-8555, Japan; d Department of Chemical and Pharmaceutical Sciences, via Giorgieri 1, 34127 Trieste, Italy

**Keywords:** crystal structure, nickel complex, di­thio­carbazate

## Abstract

In the title complex, [Ni(C_15_H_21_N_2_S_2_)_2_], the Ni^II^ atom exhibits a square-planar coordination geometry and is located on an inversion centre leading to a *trans* configuration of the *N*,*S*-chelating ligands. In the crystal, the complex mol­ecules stack at a distance of 4.6738 (3) Å along the *a* axis, which exclude any significant inter­actions between the aromatic rings.

## Related literature   

For the structures of related complexes, see: Chan *et al.* (2008 ▸); Islam *et al.* (2011 ▸, 2014 ▸); Li *et al.* (2006 ▸); Zhang *et al.* (2004 ▸). For the structure of the ligand, see: Howlader *et al.* (2015 ▸).
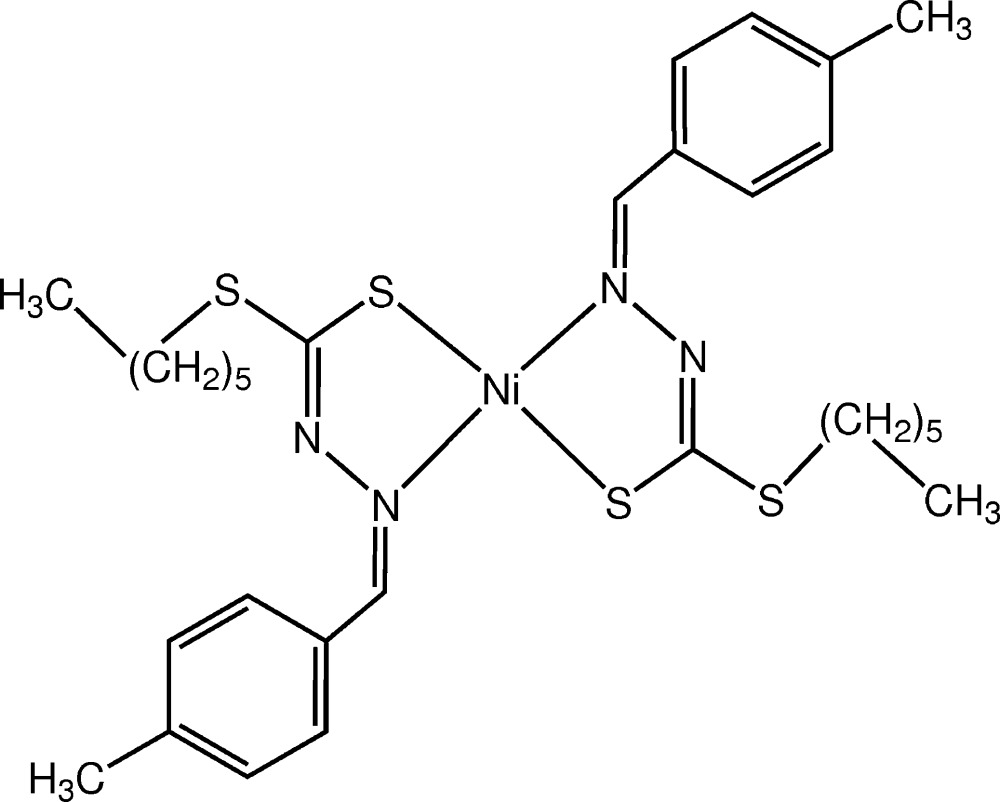


## Experimental   

### Crystal data  

[Ni(C_15_H_21_N_2_S_2_)_2_]
*M* *_r_* = 645.62
Triclinic, 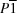

*a* = 4.6738 (3) Å
*b* = 10.5132 (5) Å
*c* = 16.4789 (8) Å
α = 86.522 (3)°
β = 84.850 (3)°
γ = 79.057 (3)°
*V* = 791.00 (7) Å^3^
*Z* = 1
Cu *K*α radiation
μ = 3.55 mm^−1^
*T* = 173 K
0.37 × 0.08 × 0.02 mm

### Data collection  

Rigaku R-AXIS RAPID diffractometer
Absorption correction: multi-scan (*ABSCOR*; Rigaku, 1995 ▸) *T*_min_ = 0.615, *T*_max_ = 0.932
9100 measured reflections
2834 independent reflections
2029 reflections with *F* ^2^ > 2σ(*F* ^2^)
*R*_int_ = 0.074

### Refinement  

*R*[*F* ^2^ > 2σ(*F* ^2^)] = 0.071
*wR*(*F* ^2^) = 0.218
*S* = 1.09
2834 reflections
180 parameters
H-atom parameters constrained
Δρ_max_ = 0.98 e Å^−3^
Δρ_min_ = −0.35 e Å^−3^

### 

Data collection: *RAPID-AUTO* (Rigaku, 2001 ▸); cell refinement: *RAPID-AUTO*; data reduction: *RAPID-AUTO*; program(s) used to solve structure: *SIR92* (Altomare *et al.*, 1994 ▸); program(s) used to refine structure: *SHELXL97* (Sheldrick, 2008 ▸); molecular graphics: *CrystalStructure* (Rigaku, 2010 ▸); software used to prepare material for publication: *CrystalStructure*.

## Supplementary Material

Crystal structure: contains datablock(s) General, I. DOI: 10.1107/S2056989015000328/ds2244sup1.cif

Structure factors: contains datablock(s) I. DOI: 10.1107/S2056989015000328/ds2244Isup2.hkl

**Fig. 1.**
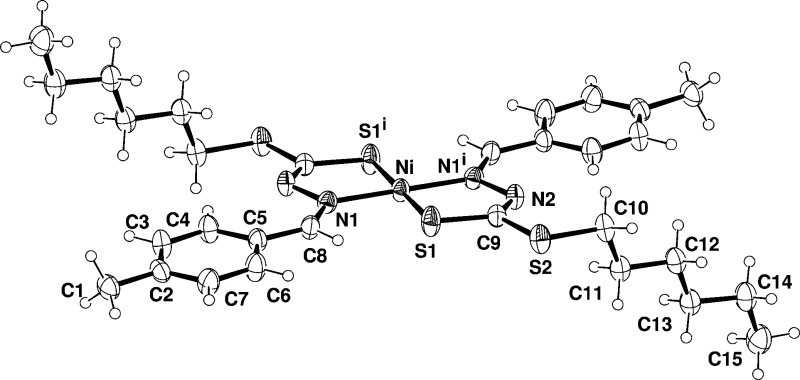
ORTEP drawing (ellipsoid probability at 50%) of the centrosymmetric NiL2 complex.

ORTEP L 2 . DOI: 10.1107/S2056989015000328/ds2244fig1.tif
*ORTEP* drawing (ellipsoid probability at 50%) of the centrosymmetric Ni*L*_2_ complex.

**Fig. 2.**
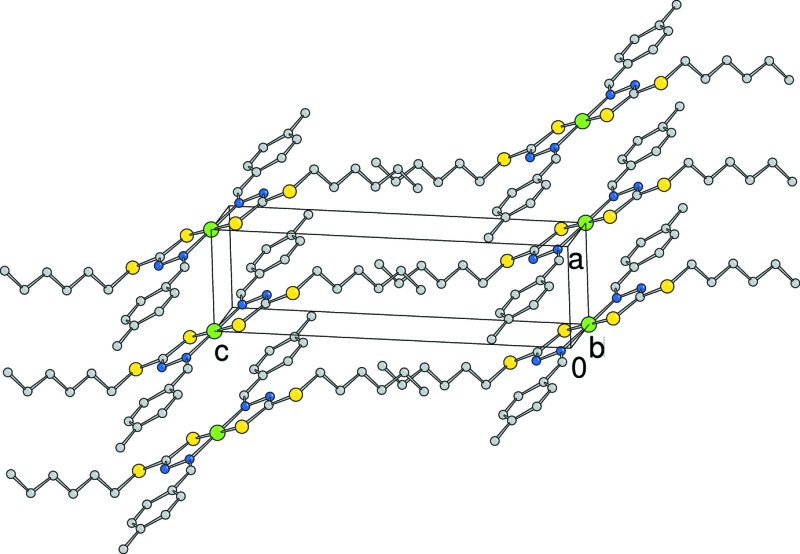
Crystal packing of the complex.

. DOI: 10.1107/S2056989015000328/ds2244fig2.tif
Crystal packing of the complex.

CCDC reference: 1035820

Additional supporting information:  crystallographic information; 3D view; checkCIF report

## Figures and Tables

**Table 1:** Selected bond lengths ()

NiN1	1.933 (3)
NiS1	2.1775 (10)

## supplementary crystallographic information

### S1. Structural commentary

The metal is located on a crystallographic inversion centre and the two Schiff bases, in their deprotonated imino thiol­ate form, act as chelating ligands to the metal centre via the azomethine nitro­gen N1 and thiol­ate sulphur S1 atoms in a trans-planar configuration as imposed by the crystal symmetry. The complex has coplanar geometry with the exception of the hexyl chains that pend hedgewise. In the complex, the Ni—S and Ni–N bond distances are of 2.1777 (11) and 1.933 (4) Å, respectively, with a S(2)—Ni—N(2) chelating angle of 86.06 (10)°. These geometrical parameters agree with those reported for similar nickel complexes either when ligands assume a *trans* (Islam, *et al.*, 2011; Islam, *et al.*, 2014; Zhang, *et al.*, 2004) or a *cis* configuration (Chan, *et al.*, 2008; Li, *et al.*, 2006). The ligand, recently reported (Howlader, *et al.*, 2015), underwent rotation about the C9—N2 by 180° in order to allow the N,S chelating behavior towards the metal. Upon coordination some salient features are observed with respect to the free ligand, and the most significant are an elongation of the C(9)—S(1) bond length (1.720 (4) Å in NiL_2_ that must be compared to 1.670 (3) Å in HL, thus validating the coordination with deprotonated thiol­ate sulphur atom. Correspondingly the N(2)—C(9) bond length, of 1.335 (3) Å, shortens to 1.270 (6) Å in the NiL_2_ complex, while the N(1)—N(2) bond length of 1.375 (3) Å in HL is slightly elongated in the complex (1.426 (5) Å, Table 1).

### S2. Supra­molecular features

The complexes stack at a distance of 4.6738 (3) Å (axis *a*), which exclude any significant inter­actions between the aromatic rings.

### S3. Synthesis and crystallization

A solution of Ni(CH_3_COO)_2_.4H_2_O (0.06 g, 0.25 mmol, 8 mL methanol) was added to a solution of the ligand, S-hexyl (E)-3-(4-methyl­benzyl­idene)di­thio­carbazate, (0.15 g, 0.5 mmol, 10 mL methanol ). The resulting mixture was stirred at room temperature for four hours. A dark reddish brown precipitate was formed, filtered off, washed with methanol and dried in vacuo over anhydrous CaCl_2_. Dark reddish brown single crystals, suitable for X-ray diffraction, of the compound were obtained by slow evaporation from a mixture of chloro­form and aceto­nitrile (1:1) after 7 days. M.P. 374 K.

### S4. Refinement

Crystal data, data collection and structure refinement details are summarized in Table 1. All H atoms were located geometrically and treated as riding atoms, with C—H = 0.95–0.99 Å, and with *U*ĩso~(H) = 1.2*U*~eq~(C) or 1.5*U*~eq~(C) for methyl H atoms.

### Crystal data

**Table:**

[Ni(C_15_H_21_N_2_S_2_)_2_]	*Z* = 1
*M**_r_* = 645.62	*F*(000) = 342.00
Triclinic, *P*1	*D*_x_ = 1.355 Mg m^−^^3^
Hall symbol: -P 1	Cu *K*α radiation, λ = 1.54187 Å
*a* = 4.6738 (3) Å	Cell parameters from 5923 reflections
*b* = 10.5132 (5) Å	θ = 4.3–68.2°
*c* = 16.4789 (8) Å	µ = 3.55 mm^−^^1^
α = 86.522 (3)°	*T* = 173 K
β = 84.850 (3)°	Platelet, brown
γ = 79.057 (3)°	0.37 × 0.08 × 0.02 mm
*V* = 791.00 (7) Å^3^	

### Data collection

**Table:**

Rigaku R-AXIS RAPID diffractometer	2029 reflections with *F*^2^ > 2σ(*F*^2^)
Detector resolution: 10.000 pixels mm^-1^	*R*_int_ = 0.074
ω scans	θ_max_ = 68.2°
Absorption correction: multi-scan (*ABSCOR*; Rigaku, 1995)	*h* = −5→5
*T*_min_ = 0.615, *T*_max_ = 0.932	*k* = −12→12
9100 measured reflections	*l* = −19→19
2834 independent reflections	

### Refinement

**Table:**

Refinement on *F*^2^	Secondary atom site location: difference Fourier map
*R*[*F*^2^ > 2σ(*F*^2^)] = 0.071	Hydrogen site location: inferred from neighbouring sites
*wR*(*F*^2^) = 0.218	H-atom parameters constrained
*S* = 1.09	*w* = 1/[σ^2^(*F*_o_^2^) + (0.1314*P*)^2^] where *P* = (*F*_o_^2^ + 2*F*_c_^2^)/3
2834 reflections	(Δ/σ)_max_ < 0.001
180 parameters	Δρ_max_ = 0.98 e Å^−^^3^
0 restraints	Δρ_min_ = −0.35 e Å^−^^3^
Primary atom site location: structure-invariant direct methods	

### Special details

**Table:**

Refinement. Refinement was performed using all reflections. The weighted *R*-factor (*wR*) and goodness of fit (*S*) are based on *F*^2^. *R*-factor (gt) are based on *F*. The threshold expression of *F*^2^ > 2.0 σ(*F*^2^) is used only for calculating *R*-factor (gt).

### Fractional atomic coordinates and isotropic or equivalent isotropic displacement parameters (Å^2^)

**Table:**

	*x*	*y*	*z*	*U*_iso_*/*U*_eq_	
Ni	1.0000	1.0000	1.0000	0.0372 (4)	
S1	1.1112 (3)	0.83443 (10)	0.92242 (6)	0.0516 (4)	
S2	1.4585 (2)	0.80909 (9)	0.76635 (6)	0.0479 (4)	
N1	0.7454 (7)	0.9180 (3)	1.07644 (18)	0.0365 (8)	
N2	1.3765 (7)	1.0201 (3)	0.85050 (18)	0.0405 (8)	
C1	−0.0675 (10)	0.4837 (4)	1.2473 (3)	0.0514 (11)	
H1A	−0.0056	0.4630	1.3025	0.077*	
H1B	−0.0491	0.4035	1.2184	0.077*	
H1C	−0.2717	0.5288	1.2503	0.077*	
C2	0.1222 (9)	0.5695 (4)	1.2022 (2)	0.0420 (10)	
C3	0.1476 (9)	0.6854 (4)	1.2321 (3)	0.0489 (11)	
H3	0.0431	0.7105	1.2825	0.059*	
C4	0.3184 (9)	0.7676 (4)	1.1923 (2)	0.0455 (10)	
H4	0.3281	0.8471	1.2154	0.055*	
C5	0.4769 (8)	0.7344 (4)	1.1181 (2)	0.0390 (9)	
C6	0.4513 (10)	0.6168 (4)	1.0883 (3)	0.0522 (12)	
H6	0.5555	0.5913	1.0380	0.063*	
C7	0.2807 (10)	0.5349 (4)	1.1288 (2)	0.0511 (11)	
H7	0.2718	0.4547	1.1063	0.061*	
C8	0.6638 (9)	0.8089 (4)	1.0680 (2)	0.0418 (10)	
H8	0.7429	0.7691	1.0186	0.050*	
C9	1.3189 (8)	0.9071 (4)	0.8487 (2)	0.0370 (9)	
C10	1.6632 (9)	0.9110 (4)	0.7013 (3)	0.0469 (11)	
H10A	1.7787	0.9530	0.7355	0.056*	
H10B	1.8022	0.8559	0.6632	0.056*	
C11	1.4718 (9)	1.0153 (4)	0.6522 (2)	0.0463 (10)	
H11A	1.3404	1.0736	0.6903	0.056*	
H11B	1.3482	0.9738	0.6203	0.056*	
C12	1.6461 (9)	1.0959 (4)	0.5943 (2)	0.0467 (10)	
H12A	1.7684	1.1383	0.6262	0.056*	
H12B	1.7784	1.0378	0.5563	0.056*	
C13	1.4508 (9)	1.1991 (4)	0.5453 (2)	0.0488 (11)	
H13A	1.3236	1.1563	0.5154	0.059*	
H13B	1.3227	1.2580	0.5837	0.059*	
C14	1.6143 (10)	1.2790 (4)	0.4849 (3)	0.0545 (12)	
H14A	1.7445	1.2204	0.4468	0.065*	
H14B	1.7386	1.3235	0.5147	0.065*	
C15	1.4131 (11)	1.3796 (5)	0.4361 (3)	0.0670 (14)	
H15A	1.3058	1.3358	0.4015	0.100*	
H15B	1.5293	1.4337	0.4019	0.100*	
H15C	1.2742	1.4342	0.4735	0.100*	

### Atomic displacement parameters (Å^2^)

**Table:**

	*U*^11^	*U*^22^	*U*^33^	*U*^12^	*U*^13^	*U*^23^
Ni	0.0441 (7)	0.0337 (6)	0.0351 (5)	−0.0150 (4)	0.0033 (4)	0.0027 (4)
S1	0.0706 (9)	0.0392 (6)	0.0472 (6)	−0.0254 (6)	0.0172 (6)	−0.0036 (5)
S2	0.0571 (8)	0.0331 (6)	0.0520 (6)	−0.0121 (5)	0.0142 (5)	−0.0052 (4)
N1	0.044 (2)	0.0325 (16)	0.0337 (16)	−0.0097 (15)	0.0010 (14)	−0.0006 (13)
N2	0.045 (2)	0.0378 (18)	0.0370 (17)	−0.0105 (16)	0.0085 (15)	0.0032 (14)
C1	0.047 (3)	0.047 (2)	0.061 (3)	−0.016 (2)	−0.001 (2)	0.013 (2)
C2	0.034 (2)	0.042 (2)	0.050 (2)	−0.0104 (18)	−0.0010 (18)	0.0111 (18)
C3	0.051 (3)	0.044 (2)	0.051 (2)	−0.013 (2)	0.016 (2)	−0.0023 (19)
C4	0.053 (3)	0.034 (2)	0.049 (2)	−0.015 (2)	0.012 (2)	−0.0064 (17)
C5	0.040 (2)	0.039 (2)	0.038 (2)	−0.0142 (18)	0.0024 (18)	0.0049 (16)
C6	0.071 (3)	0.045 (2)	0.044 (2)	−0.027 (2)	0.013 (2)	−0.0083 (19)
C7	0.068 (3)	0.043 (2)	0.048 (2)	−0.027 (2)	0.004 (2)	−0.0037 (18)
C8	0.050 (3)	0.044 (2)	0.0337 (19)	−0.017 (2)	0.0048 (18)	−0.0028 (16)
C9	0.036 (2)	0.037 (2)	0.036 (2)	−0.0065 (18)	0.0024 (17)	0.0012 (16)
C10	0.049 (3)	0.039 (2)	0.050 (2)	−0.010 (2)	0.016 (2)	−0.0037 (18)
C11	0.050 (3)	0.044 (2)	0.045 (2)	−0.014 (2)	0.007 (2)	−0.0020 (18)
C12	0.048 (3)	0.043 (2)	0.049 (2)	−0.016 (2)	0.009 (2)	−0.0037 (18)
C13	0.051 (3)	0.050 (2)	0.047 (2)	−0.017 (2)	0.007 (2)	−0.0016 (19)
C14	0.061 (3)	0.047 (2)	0.056 (3)	−0.019 (2)	0.011 (2)	−0.001 (2)
C15	0.079 (4)	0.061 (3)	0.063 (3)	−0.026 (3)	0.001 (3)	0.007 (2)

### Geometric parameters (Å, º)

**Table:**

Ni—N1	1.933 (3)	C6—C7	1.385 (5)
Ni—N1^i^	1.933 (3)	C6—H6	0.9500
Ni—S1	2.1775 (10)	C7—H7	0.9500
Ni—S1^i^	2.1775 (10)	C8—H8	0.9500
S1—C9	1.720 (4)	C10—C11	1.519 (5)
S2—C9	1.757 (4)	C10—H10A	0.9900
S2—C10	1.811 (4)	C10—H10B	0.9900
N1—C8	1.294 (5)	C11—C12	1.521 (5)
N1—N2^i^	1.425 (4)	C11—H11A	0.9900
N2—C9	1.269 (5)	C11—H11B	0.9900
N2—N1^i^	1.425 (4)	C12—C13	1.521 (6)
C1—C2	1.501 (5)	C12—H12A	0.9900
C1—H1A	0.9800	C12—H12B	0.9900
C1—H1B	0.9800	C13—C14	1.513 (5)
C1—H1C	0.9800	C13—H13A	0.9900
C2—C3	1.371 (6)	C13—H13B	0.9900
C2—C7	1.391 (6)	C14—C15	1.517 (6)
C3—C4	1.383 (5)	C14—H14A	0.9900
C3—H3	0.9500	C14—H14B	0.9900
C4—C5	1.399 (5)	C15—H15A	0.9800
C4—H4	0.9500	C15—H15B	0.9800
C5—C6	1.387 (5)	C15—H15C	0.9800
C5—C8	1.452 (5)		
			
N1—Ni—N1^i^	180.00 (14)	N2—C9—S1	125.9 (3)
N1—Ni—S1	93.96 (9)	N2—C9—S2	120.3 (3)
N1^i^—Ni—S1	86.04 (9)	S1—C9—S2	113.9 (2)
N1—Ni—S1^i^	86.04 (9)	C11—C10—S2	113.5 (3)
N1^i^—Ni—S1^i^	93.96 (9)	C11—C10—H10A	108.9
S1—Ni—S1^i^	180.0	S2—C10—H10A	108.9
C9—S1—Ni	95.86 (13)	C11—C10—H10B	108.9
C9—S2—C10	103.09 (19)	S2—C10—H10B	108.9
C8—N1—N2^i^	113.8 (3)	H10A—C10—H10B	107.7
C8—N1—Ni	126.3 (3)	C10—C11—C12	113.2 (3)
N2^i^—N1—Ni	119.9 (2)	C10—C11—H11A	108.9
C9—N2—N1^i^	111.9 (3)	C12—C11—H11A	108.9
C2—C1—H1A	109.5	C10—C11—H11B	108.9
C2—C1—H1B	109.5	C12—C11—H11B	108.9
H1A—C1—H1B	109.5	H11A—C11—H11B	107.8
C2—C1—H1C	109.5	C13—C12—C11	112.4 (3)
H1A—C1—H1C	109.5	C13—C12—H12A	109.1
H1B—C1—H1C	109.5	C11—C12—H12A	109.1
C3—C2—C7	117.1 (4)	C13—C12—H12B	109.1
C3—C2—C1	121.3 (4)	C11—C12—H12B	109.1
C7—C2—C1	121.7 (4)	H12A—C12—H12B	107.9
C2—C3—C4	123.0 (4)	C14—C13—C12	114.4 (4)
C2—C3—H3	118.5	C14—C13—H13A	108.7
C4—C3—H3	118.5	C12—C13—H13A	108.7
C3—C4—C5	120.4 (4)	C14—C13—H13B	108.7
C3—C4—H4	119.8	C12—C13—H13B	108.7
C5—C4—H4	119.8	H13A—C13—H13B	107.6
C6—C5—C4	116.3 (3)	C13—C14—C15	113.0 (4)
C6—C5—C8	116.0 (3)	C13—C14—H14A	109.0
C4—C5—C8	127.8 (4)	C15—C14—H14A	109.0
C7—C6—C5	122.9 (4)	C13—C14—H14B	109.0
C7—C6—H6	118.6	C15—C14—H14B	109.0
C5—C6—H6	118.6	H14A—C14—H14B	107.8
C6—C7—C2	120.3 (4)	C14—C15—H15A	109.5
C6—C7—H7	119.9	C14—C15—H15B	109.5
C2—C7—H7	119.9	H15A—C15—H15B	109.5
N1—C8—C5	133.6 (4)	C14—C15—H15C	109.5
N1—C8—H8	113.2	H15A—C15—H15C	109.5
C5—C8—H8	113.2	H15B—C15—H15C	109.5
			
C7—C2—C3—C4	0.8 (6)	C4—C5—C8—N1	2.7 (8)
C1—C2—C3—C4	−179.6 (4)	N1^i^—N2—C9—S1	1.8 (5)
C2—C3—C4—C5	−0.2 (7)	N1^i^—N2—C9—S2	−177.5 (2)
C3—C4—C5—C6	0.0 (6)	Ni—S1—C9—N2	2.5 (4)
C3—C4—C5—C8	179.5 (4)	Ni—S1—C9—S2	−178.24 (18)
C4—C5—C6—C7	−0.2 (7)	C10—S2—C9—N2	−0.4 (4)
C8—C5—C6—C7	−179.8 (4)	C10—S2—C9—S1	−179.8 (2)
C5—C6—C7—C2	0.8 (7)	C9—S2—C10—C11	−77.3 (3)
C3—C2—C7—C6	−1.0 (6)	S2—C10—C11—C12	−176.9 (3)
C1—C2—C7—C6	179.3 (4)	C10—C11—C12—C13	179.6 (3)
N2^i^—N1—C8—C5	−1.2 (7)	C11—C12—C13—C14	−178.2 (3)
Ni—N1—C8—C5	−179.8 (3)	C12—C13—C14—C15	179.1 (4)
C6—C5—C8—N1	−177.7 (4)		

Symmetry code: (i) −*x*+2, −*y*+2, −*z*+2.
