# Supplementary figures and images for: Crystal structure of bis­[S-hexyl 3-(4-methyl­benzyl­idene)di­thio­carbazato-κ2 N 3,S]nickel(II)
Source: Acta Crystallogr E Crystallogr Commun. 2015 Jan 14;71(Pt 2):m26–7. doi: 10.1107/S2056989015000328 (PMC4384570; doi:10.1107/S2056989015000328)

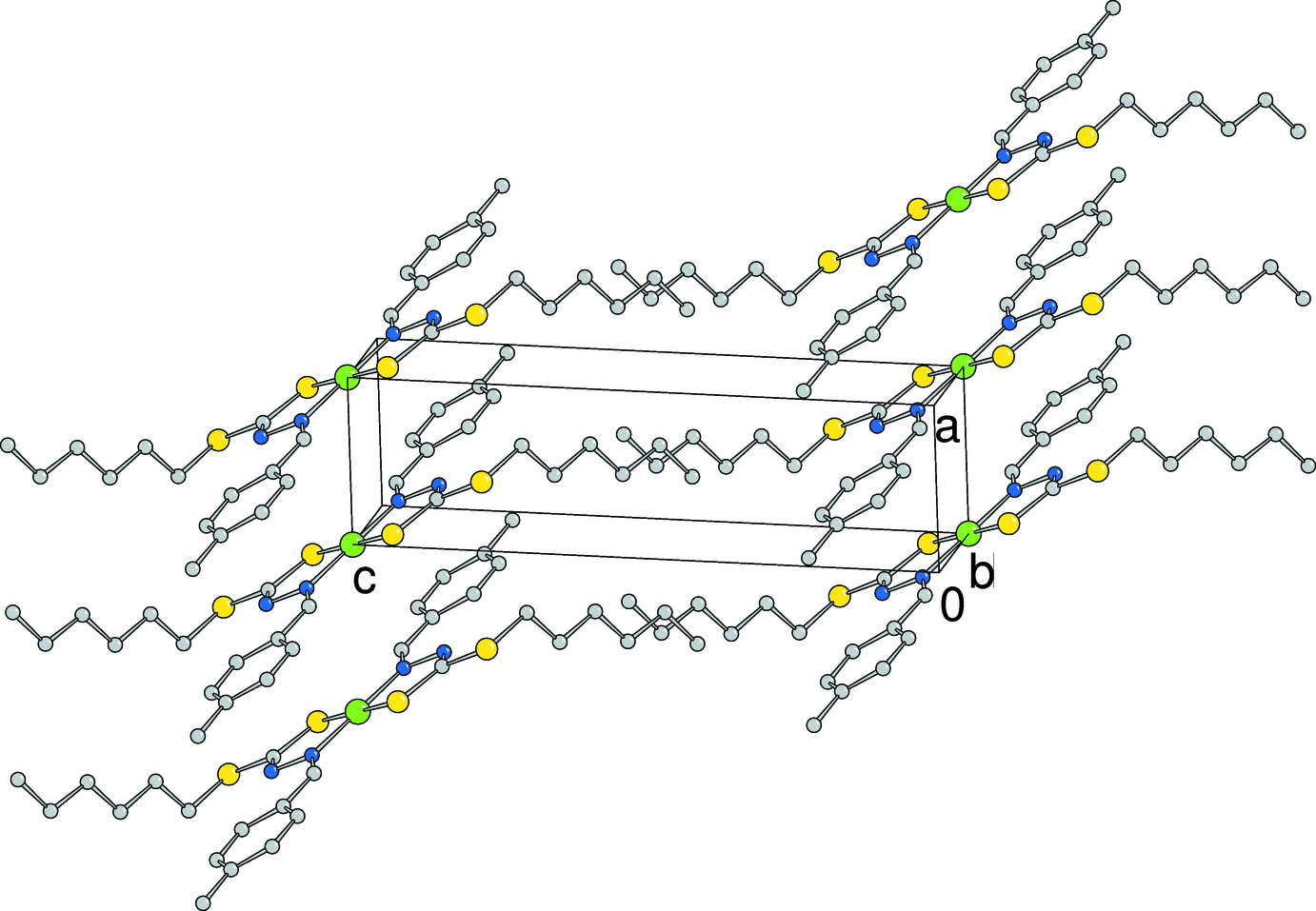

Supplement: Supplementary file 4 [file e-71-00m26-fig2.tif]
